# The alternative 3′ splice site of *GPNMB* may promote neuronal survival after neonatal hypoxic–ischemic encephalopathy injury

**DOI:** 10.1002/ibra.12056

**Published:** 2022-08-09

**Authors:** Guo‐Jiao Chen, Shan‐Shan Yan, Jing‐Han Zhang, Ji Zhang, Isaac Bul Deng, Rong He

**Affiliations:** ^1^ Bioinformatics Center Kunming Medical University Kunming Yunnan China; ^2^ Southwest Medical University Luzhou Sichuan China; ^3^ Center for Epogenetics and Induced Pluripotent Stem Cells, Kennedy Krieger Institute Johns Hopkins University Baltimore USA

**Keywords:** alternative splicing, gene sequencing, *GPNMB*, neonatal hypoxic–ischemic encephalopathy, neuronal survival

## Abstract

This study aimed to decipher the effect of glycoprotein nonmetastatic melanoma protein B (*GPNMB*) on neonatal hypoxic–ischemic encephalopathy (NHIE) and its potential molecular mechanism. The hypoxic–ischemic (HI) model was established in 7‐day‐old rats, and then, Zea‐Longa scores and Nissl staining were performed to measure brain damage post‐HI. In addition, gene sequencing was used to detect the differential expression genes (DEGs), and then, Gene Ontology and Kyoto Encyclopedia of Genes and Genomes databases were used to determine the function of DEGs. Furthermore, an oxygen–glucose deprivation (OGD) model was developed in SY5Y cells and human fetal neurons, and then, the level of *GPNMB* was verified by quantitative real‐time polymerase chain reaction. In addition, methyl thiazolyl tetrazolium and cell counting kit‐8 assays were applied after *GPNMB* interference. Finally, the alternative splicing of *GPNMB* expression was analyzed using Splice Grapher software. The results indicated that HI induced marked neurological impairment and neuron injury in rats. Also, *GPNMB* was the most obviously upregulated gene in DEGs. Additionally, *GPNMB* was upregulated significantly in SY5Y and fetal neurons after OGD, and GPNMB‐si promoted an increase in cell viability and number. Moreover, we found that the *GPNMB* alternative splicing type was the Alternative 3′ splice site, with the alternative splicing site in 143382985:143404102. Herein, *GPNMB* promotes a crucial regulatory mechanism with alternative splicing for neuronal survival after NHIE.

## Graphical abstract

Glycoprotein nonmetastatic melanoma protein B (*GPNMB*) was the most obviously upregulated gene in differential expression genes post hypoxic–ischemic. Additionally, *GPNMB* was upregulated significantly in SY5Y and fetal neurons after oxygen–glucose deprivation, and *GPNMB*‐si promoted an increase in cell viability and number. Moreover, we found that the GPNMB alternative splicing type was the alternative 3′ splice site, with the alternative splicing site in 143382985:143404102.

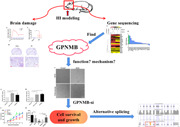

## 1 INTRODUCTION

Neonatal hypoxic–ischemic encephalopathy (NHIE) is a severe perinatal complication caused by perinatal asphyxia.^1^ Studies have shown that the incidence of NHIE in newborns is about 1.5 per 1000.^2^ It is widely known that NHIE can cause neonatal death and severe morbidity, such as cerebral palsy, epilepsy, sensory, and cognitive and learning disabilities.^3^, ^4^ Cerebral hypoxia–ischemia (HI) can trigger a series of responses, including energy failure, production of nitric oxide, excitatory toxic amino acid release, oxidative stress, and inflammatory responses, and finally result in apoptosis and necrosis.^4^ NHIE adversely affects the intellectual development of children and poses a great burden to families and society. However, effective therapeutic measures and molecular mechanisms are yet to be improved, so it is of great significance to seek new therapeutic targets for NHIE.

The glycoprotein nonmetastatic melanoma protein B (GPNMB) functions as a membrane‐binding surface receptor, a melanosome‐associated protein, a soluble ligand, or an adhesion molecule; it is highly expressed in various cell types and manages homeostasis in a variety of tissues. It is known that *GPNMB* plays a crucial role in different kinds of diseases, such as various cancers like glioma,^5^ breast cancer,^6^ hepatocellular carcinoma,^7^ and gastric cancer,^8^ chronic obstructive pulmonary disease,^9^ obesity,^10^ and so on. Moreover, it has been shown that the *GPNMB* extracellular fragment has the ability to interact with a variety of receptors, such as CD44, Na^+^, K^+^‐ATPase (NKA), epidermal, and vascular endothelial growth factor receptors.^11^ More recently, studies have found that GPNMB is commonly expressed in the brain of rats.^12^ Besides, some evidence suggests that GPNMB expression is upregulated in the spinal cord of amyotrophic lateral sclerosis (ALS) patients.^13^ Similarly, GPNMB expression was also found to be increased in animal models of cerebral ischemia injury. It is associated with the reduction of cerebral infarction volume,^14^ suggesting that GPNMB plays a vital role in neurological damage. However, with regard to NHIE, there are few studies on the effect of GPNMB.

Gene sequencing^15^ technology has been widely used in a large number of biology and medicine studies in recent years, which provides crucial theoretical and practical values for diseases research. The technology can provide large‐scale, high‐throughput information, as well as integrate a range of biological data.^16^ Therefore, it can provide more comprehensive knowledge in the research of disease mechanisms and lay a reliable foundation for further mechanism research.

Herein, in the present study, the NHIE model was established in vivo and in vitro, and gene sequencing was used to screen out differential expression genes (DEGs). Afterward, *GPNMB* interference was performed to identify its role in vitro, and the potential molecular mechanism was investigated by alternative splicing.

## 2 MATERIALS AND METHODS

### 2.1 Animal care

Sprague–Dawley (SD) rats used in this study were 7 days old and were obtained from the Animal Center of Kunming Medical University. The care and feeding of rats in this experiment were in conformity with the provisions of the Chinese Laboratory Animal Protection and the Animal Care and Welfare Committee of Kunming Medical University (No. Kmmu2019038). All experiments were consistent with the Guide for the Care and Use of Laboratory Animal published by the United States National Institutes of Health. All animals were kept in a standard environment, including a 12 h light/dark cycle, 45%–50% humidity, room temperature at 21–25°C, and sufficient food and water.

### 2.2 Animal grouping and establishment of a neonatal hypoxic–ischemic encephalopathy model

All the rats were randomly divided into a Sham‐operated (Sham) group and a hypoxic–ischemic group. The establishment of the neonatal hypoxic–ischemic encephalopathy model was performed as previously described.^17^ In brief, 7‐day‐old SD rats (weighing 12–15 g) were anesthetized with 3% isoflurane, and a longitudinal incision about 1 cm was performed in the middle of the neck. The right common carotid artery was carefully separated and blocked by electrocoagulation to establish ischemia. Subsequently, the subcutaneous tissue and skin were sutured. After the operation, rats were returned to cages to recover for 1 h. Finally, these rats were placed in a hypoxia chamber (8% oxygen and 92% nitrogen gas mixture) for 2 h to induce hypoxia. The rats in the Sham group were subjected to anesthesia, and the common carotid artery was exposed without arterial occlusion and hypoxia.

### 2.3 Zea‐Longa scores

The severity of the neurological injury was evaluated according to Zea‐Longa scores, ranging from 0 to 4 (the normal score is 0; the maximum defect score is 4). The neurobehavioral scores of the Sham group and the HI group were determined at 1, 2, 3, 4, 5, 6, and 7 days post‐HI. The higher the scores, the more severe the damage. Three experimenters evaluated each rat in a blinded manner; these three members did not participate in the creation of the model and were unaware of the experimental groupings.

### 2.4 Tissue harvest

After the nerve behavior test, animals were anesthetized and perfused with physiological saline. For gene analysis, the brains of rats were isolated, the cortex was collected, and then stored at −80°C. For Nissl's staining, brains were fixed with 4% paraformaldehyde, buried in paraffin, and cut into 5 µm slides.

### 2.5 Nissl staining

After the NHIE model was established, Nissl staining was used to detect brain injury. In brief, the brain slides were dewaxed in xylene and dehydrated in gradient ethanol. Then, Cresyl Violet Stain solution was added to immerse the slides in the dark at room temperature. Subsequently, the slides were dripped with Nissl differentiation solution after rinsing with distilled water. Afterward, the slides were dehydrated with ethanol and mounted with neutral resin. Finally, photographs were taken using a high‐power optical microscope. The brain injury area was quantified as infarct volume in the right hemisphere = total cortex volume in the left hemisphere of the same brain − noninfarcted cortex volume in the right hemisphere of the same brain.^18^

### 2.6 Gene sequencing

Gene sequencing was performed according to the previously described procedure, with certain modifications.^19^, ^20^ First, the total RNA was abstracted from brain tissues, and then the quality of RNA was tested using a microplate reader. After the sample was qualified, a complementary DNA (cDNA) library was constructed. After that, Qubit2.0 and Agilent 2100 were applied to determine the concentration and insert size of the library, which was quantified using the quantitative real‐time polymerase chain reaction (qRT‐PCR) method. Next, after the library qualified, the sequencing was completed by Beijing Biomark Biotechnology Co., Ltd. Finally, for testing of DEGs, the screening criteria were set as fold change (FC) ≥ two and false discovery rate < 0.05.

### 2.7 Bioinformatics analysis

The Gene Ontology (GO) database (http://www.geneontology.ogy.org/) was used to analyze the biological process (BP) of DEGs. Besides, pathway enrichment analysis of DEGs was performed using the Kyoto Encyclopedia of Genes and Genomes (KEGGs) database (http://www.genome.jp/kegg/pathway.html) or DAVID Bioinformatics Resources (https://david.ncifcrf.gov/).

### 2.8 SY5Y cell culture

SY5Y cells were obtained from the GeneCopoeia company and thawed at 37°C. Then, the cell suspension was sucked into a sterile 15 ml centrifuge tube and mixed by adding prewarmed Dulbecco's modified Eagle's medium (DMEM)/F‐12 complete medium. After centrifugation (1500 rpm) for 4 min, the supernatant was removed. Afterward, a complete fresh medium (DMEM/F12 + 10% fetal bovine serum [FBS]) was added to the centrifuge tube to obtain a cell suspension. After mixing, SY5Y cells were transferred into a 25 cm^2^ culture flask and placed in an incubator at 37°C, 5% CO_2_. The medium was replaced once the next day. Once the cells had grown up to about 80% of the bottom of the flask, the cells could be passaged. The cell morphology was determined using an inverted microscope (NikonT1‐SM [TiE]) and quantified using Image‐Pro Plus 6.0 software.

### 2.9 Cultures of human fetal cortical neurons

Human fetal brain tissue was collected from an aborted 29‐day‐old fetus, which was obtained from the First Affiliated Hospital of Kunming Medical University (Approval No. 2015‐9). The cortexes were added to the culture of primary cortical neurons.^17^ In brief, the cerebral cortexes were isolated, and 0.25% trypsin was used to digest tissues for 10 min at 37°C to obtain a cell suspension; after that, 10% FBS was used to stop cell digestion. After centrifugation for 10 min at 1000 rpm, the cells were collected and resuspended using a fresh medium. After incubation in 5% CO_2_ at 37°C for 4 h, cells were cultured with Neurobasal + B27 medium. Then, half of the medium was refreshed every 3 days. On Day 6 of the cell culture, the cells were observed to be in good condition and identified whether they were neurons based on cell morphology.

### 2.10 Screening and validation of effective interference fragments

First, according to the NCBI access *GPNMB* gene sequence, three RNA interference (RNAi) fragments and a random garbage control fragment were designed by Guangzhou Ruibo company. Next, SY5Y cells were inoculated in a six‐well plate and cultured in a 5% CO_2_ incubation box at 37°C for 24 h. The effective RNAi was determined after the cell fusion rate reached 40%. On the basis of the operation manual, the cultured SY5Y cells were randomly divided into six groups (three holes cells for each group), including Normal, Reagent, NC, *GPNMB*‐F1, *GPNMB*‐F2, and *GPNMB*‐F3 groups. The interference RNA frozen powder was centrifuged (12,000 rpm) for 1 min. The mixture with small interfering RNA (siRNA) fragments was added to the culture medium, qRT‐PCR was used to verify the interference effect, and the most effective interfering fragment was selected for the following experiment.

### 2.11 Transfection of effective interference fragments

After SY5Y cells were cultured for 5 days, cells were randomly divided into normal, oxygen–glucose deprivation (OGD), OGD + reagent, OGD + NC, and OGD + *GPNMB*‐si groups, three holes cells for each group. Cell morphology was viewed at the live‐cell workstation. Then, according to the instructions provided in the manual, the interference RNA transfection system was configured and poured into each hole and then kept for 15–30 min at room temperature. The culture medium was sucked for 1 ml before adding the reflection system. After 24 h, 1 ml of medium was added to the culture medium, and the morphology of the cells was photographed using a microscope after 48 h. To count the number of cells, we randomly chose five fields of view (×200) in a well.

### 2.12 Establishment of a model of OGD

The OGD model was constructed as described previously.^21^ First of all, the cell culture medium was gently aspirated, and then well‐differentiated SY5Y cells and fetal neurons were washed and resuspended with DMEM medium without glucose. Afterward, cells were transferred to a chamber (Thermo Fisher Scientific) for 1 h to induce hypoxia, and the following parameters were set: 0% oxygen, 95% nitrogen, 5% carbon dioxide, and temperature at 37°C. Then, the sugar‐free medium was replaced with an equal amount of DMEM/F12 medium containing 10% FBS or a special neuron medium and cells were placed back into an incubator (95% air + 5% CO_2_ mixed gas at 37°C) for 24 h.

### 2.13 Methyl thiazolyl tetrazolium assay

The methyl thiazolyl tetrazolium (MTT) assay was applied to determine the cell proliferation, which was performed following the protocols of Ma et al.^22^ First, cells were inoculated at a density of 1 × 10^5^ cells per well in a 96‐well plate. Then, 20 μl of MTT solution (Sigma) was added dropwise to each well after the establishment of OGD and then held at 37°C for 4 h in the dark. In addition, the culture medium with MTT was aspirated, and a 100 μl dimethyl sulfoxide (Sigma) solution was added to the well. After that, the plate was placed on a shaker and agitated for 2–5 min. Finally, the optical density (OD) was determined using a microplate reader (Bio‐Rad) to calculate cell viability at 570 nm.

### 2.14 Cell counting kit‐8 assay

The cell counting kit‐8 (CCK‐8) assay was also used for testing cell viability in our study. In brief, first, cells were inoculated at a density of 1 × 10^5^ cells per well in a 96‐well plate and incubated in 5% CO_2_ under 37°C conditions for 24 h. Then, the CCK‐8 reagent (10 μl) was dropwise added to each well 24 h after OGD. Finally, the plate was placed on a shaker and agitated for 2–5 min after a 4 h reaction; the OD value was determined and data analysis was performed at 450 nm.

### 2.15 Quantitative real‐time polymerase chain reaction

qRT‐PCR was performed to measure the relative level of *GPNMB*. Briefly, the total RNA was isolated and reverse‐transcribed to cDNA according to the instructions provided with the kit. The primer sequences are displayed below:

*GPNMB*: Forward: *GAAATTCATCCGACGAAAC*, Reverse: *ATTGGTGGAAACAAACAGG*; *GPNMB* F1: Forward: *ACTTGGGCCTCAACTCATGG*, Reverse: *TCACTTGTGCGATGGGAACAT*; *GPNMB* F2: Forward: *TGCGAGATCACCCAGAACAC*, Reverse: *AGAGCCAGGCTTGTGTCATC*; *GPNMB* F3: Forward: *GCCCAAGCCTGTGGTATGAT*, Reverse: *CATTCCTTCACTCAGCCAAGC*; *GAPDH*: Forward: *CAAGGCTGAGAATGGGAAGC*, Reverse: *GAAGACGCCAGTAGACTCCA*.

qRT‐PCR was performed in a thermocycler: initial denaturation at 95°C for 5 min, denaturation at 95°C for 10 s, amplification at 51°C for 10 s, and extension at 72°C for 20 s. The final data were obtained by normalization to the GAPDH value using the $2^{-∆∆C_{t}}$ method.

### 2.16 Cell growth curve

Cell graphs were obtained using a high‐content imaging system.^21^ In brief, three culture plates were randomly selected, with 96 objects in each well, to obtain the original images. Then, the average number of cells was counted, and the cell growth curve was drawn according to the cells' count data every day, which was reflective of the proliferation ability of cells.

### 2.17 Alternative splicing of *GPNMB*

To elucidate the changes in *GPNMB* alternative splicing, the molecular mechanism of the alternative splicing in *GPNMB* expression was analyzed using Splice Grapher software to model the gene that predicts a new alternative splicing event.

### 2.18 Statistical analysis

All data in this study were obtained using SPSS 22.0 software and are described as mean ± standard deviation. Statistical comparisons were performed using an independent‐sample *t*‐test for two groups and one‐way analysis of variance (ANOVA) for three or more groups. In the one‐way ANOVA test, if the variance was uniform, the least significant difference test was used to compare the differences between the two groups; if the variance was uneven, the Dunnett T3 test was used for pairwise comparison. * Suggested that the *p*‐value was less than 0.05, and there was a significant difference.

## 3 RESULTS

### 3.1 Brain damage after HI

**Figure 1:**
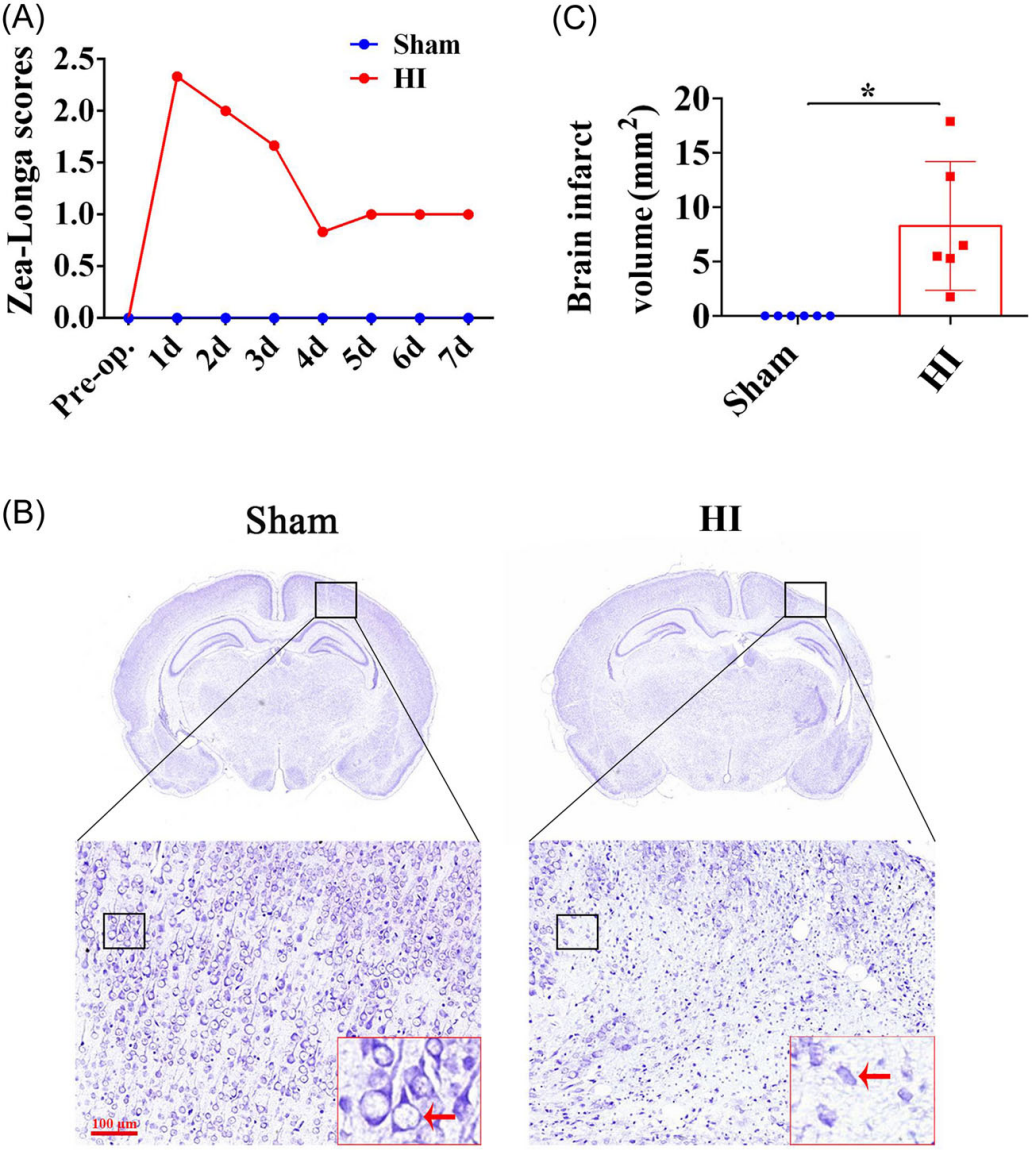
Measurement of brain injury after the establishment of the NHIE model. (A) Zea‐Longa scores in the Sham and HI groups. (B) Photographs of Nissl staining between the Sham group and the HI group. (C) Bar chart of the brain infarct volume. All data were presented as mean ± SD, *p < 0.05. HI, hypoxic–ischemic; NHIE, neonatal hypoxic–ischemic encephalopathy. [Color figure can be viewed at wileyonlinelibrary.com]

From the Zea‐Longa scores, it can be clearly seen that the scores of rats increased significantly after HI modeling, decreased gradually with time, and stabilized after 5 days (Figure 1A). The typical morphology of Nissl staining is shown in Figure 1B. In the Sham group, the neurons appeared to have a normal shape, while the neurons in the HI group were mostly stained, with irregular shapes. Besides, compared to the Sham group, the rats' brain displayed markedly brain infarct volume after HI (Figure 1C, *p* < 0.05).

### 3.2 Gene sequencing post‐HI

**Figure 2:**
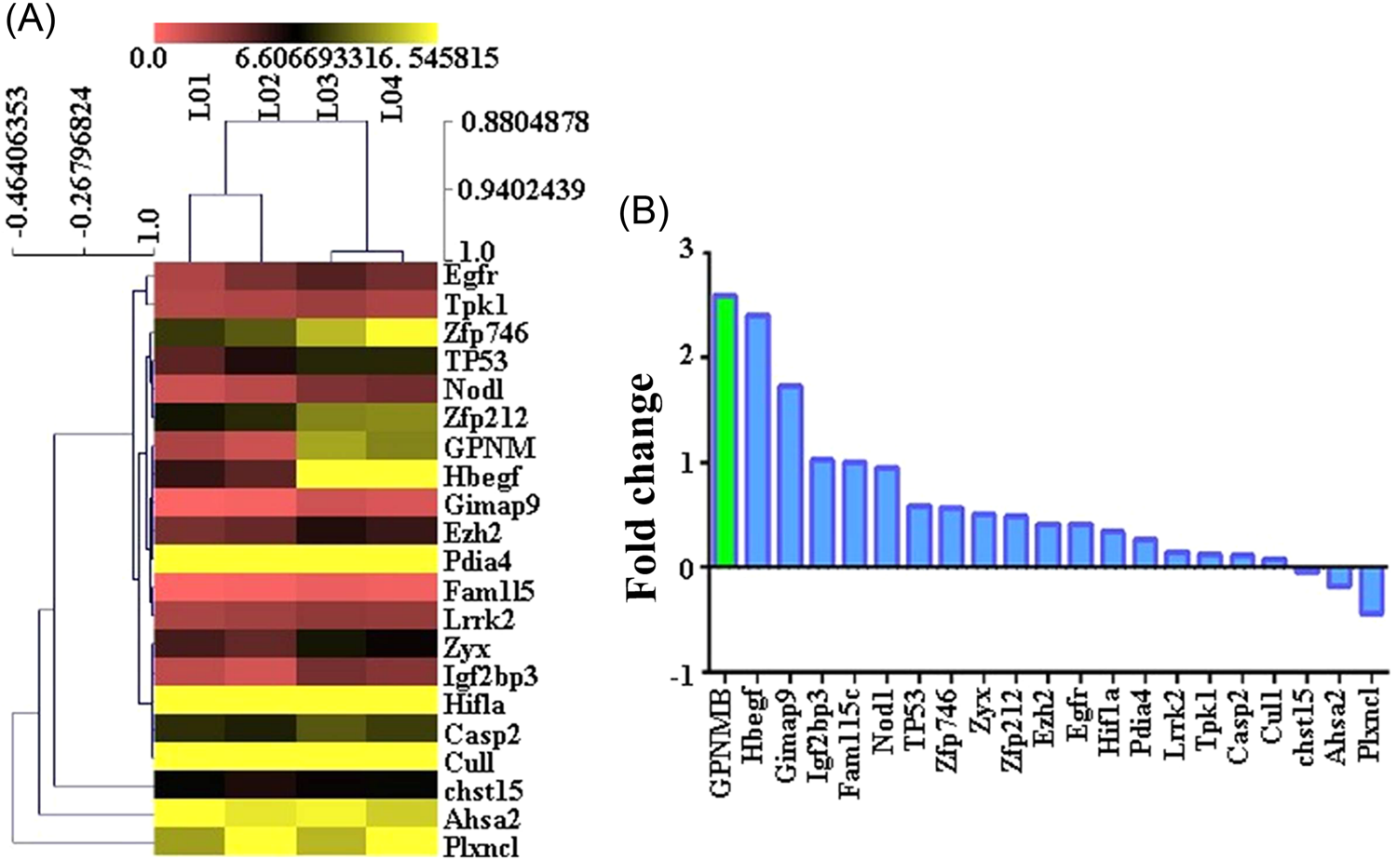
Differentially expressed gene screening. (A) A heat map of differentially expressed genes. (B) Bar chart of differentially expressed genes. [Color figure can be viewed at wileyonlinelibrary.com]

To investigate the molecular mechanism of HI, gene sequencing was performed to screen out DEGs. The DEGs were identified and displayed as a heat map (Figure 2A). The results showed that there were 18 upregulated genes (*GPNMB, Hbegf, Gimap9, Igf2bp3, Fam115c, Nod1, Tp53, Zfp746, Zyx, Zfp212, Ezh2, Egfr, Hif1a, Pdia4, Lrrk2, Tpk1, Casp2, and Cul1*) and 3 downregulated genes (*chst15, Ahsa2, and Plxncl*) in the HI group relative to the Sham group. Importantly, *GPNMB* showed the most FCs among DEGs (Figure 2B).

### 3.3 Bioinformatics analysis of differentially expressed genes

**Figure 3:**
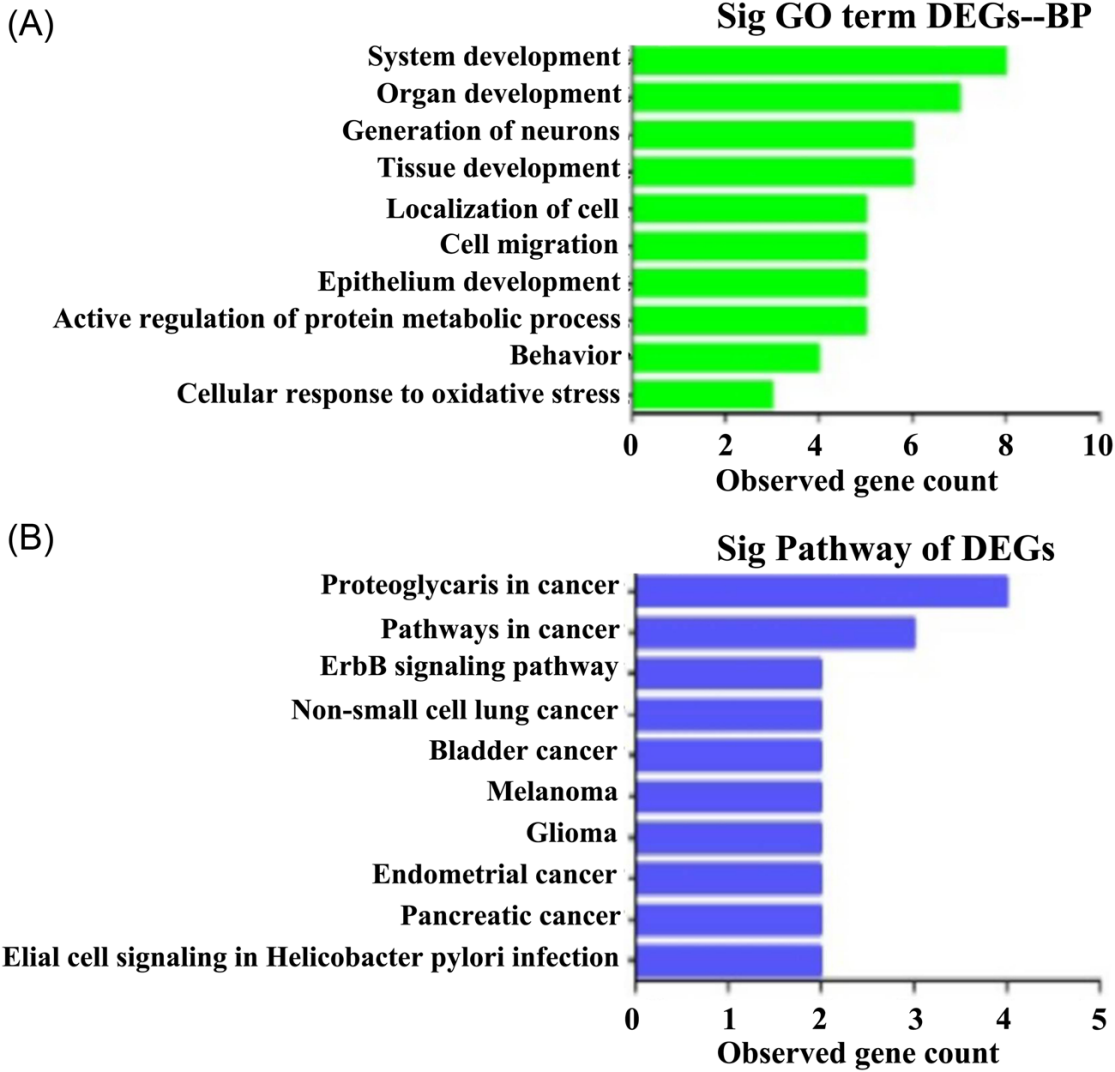
GO analysis and KEGG analysis. (A) The DEGs of the BP by GO analysis. (B) The DEGs of pathways by KEGG analysis. BP, biological process; DEGs, differentially expressed genes; GO, Gene Ontology; KEGG, Kyoto Encyclopedia of Genes and Genomes. [Color figure can be viewed at wileyonlinelibrary.com]

According to the enrichment points, we used the GO database to select the top 10 BPs, including system development, organ development, generation of neurons, tissue development, localization of cells, cell migration, epithelium development, negative regulation of the protein metabolic process, behavior, and cellular response to oxidative stress (Figure 3A). Moreover, we used the KEGG database for pathway analysis, and the top 10 signaling pathways according to the enrichment scores were as follows: proteoglycans in cancer, pathways in cancer, the ErbB signaling pathway, non‐small‐cell lung cancer, bladder cancer, melanoma, glioma, endometrial cancer, pancreatic cancer, and glial cell signaling in *Helicobacter pylori* infection (Figure 3B).

### 3.4 Verification of *GPNMB* after OGD injury

**Figure 4:**
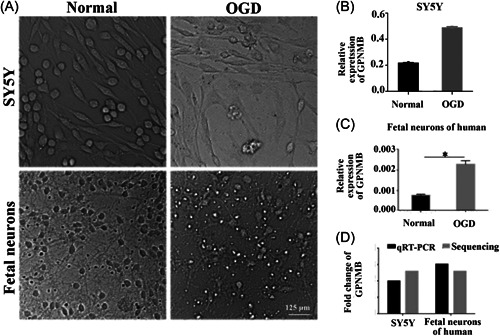
Validation of GPNMB expression in cultured fetal neurons and SY5Y cells after OGD injury. (A) The morphology of fetal neurons (down) and SY5Y cells (up) after OGD injury. Scale bar = 125 μm. (B) The *GPNMB* expression in SY5Y cells after OGD injury. (C) The *GPNMB* expression in fetal neurons of humans after OGD injury. (D) The expressional fold change of *GPNMB* in SY5Y cells and fetal neurons of humans by qRT‐PCR and sequencing, respectively. All data were presented as mean ± SD, *p < 0.05. GPNMB, glycoprotein nonmetastatic melanoma protein B; OGD, oxygen–glucose deprivation; qRT‐PCR, quantitative real‐time polymerase chain reaction.

The typical morphology of cultured human fetal neurons and SY5Y cells observed under a light microscope is shown in Figure 4A. Obviously, after OGD injury, SY5Y cells decreased, and fetal neurons became smaller, cell bodies became relatively rounded, and neurites became shorter (Figure 4A). The level of *GPNMB* was upregulated in the OGD group in SY5Y cells and fetal neurons of humans (Figure 4B,C, *p* < 0.05). This indicates that OGD might upregulate the level of *GPNMB* in cultured fetal neurons and SY5Y cells. The expression of *GPNMB* in SY5Y cells and fetal neurons was confirmed by qRT‐PCR and sequencing, respectively. We found that the expression of *GPNMB* was increased after OGD injury, which was consistent with the sequencing data and qRT‐PCR results (Figure 4D).

### 3.5 Successful transfection of effective interference fragments

**Figure 5:**
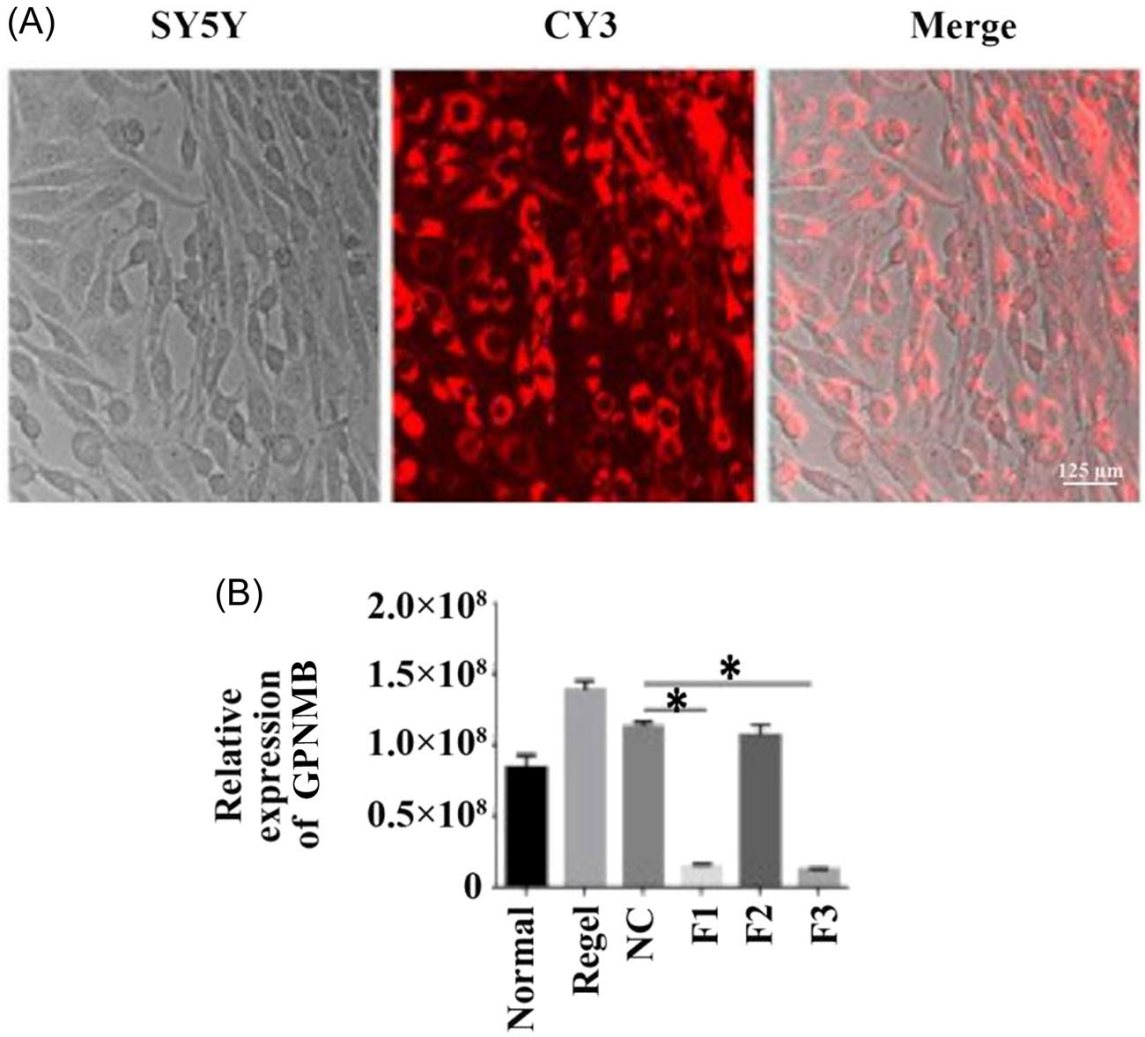
Screening, confirmation, and transfection of *GPNMB* effective interference fragments. (A) *GPNMB* effective interference fragment successfully transfected in SY5Y cells. Scale bar = 125 μm. (B) Screening of *GPNMB* interference fragments F1, F2, and F3, and the most effective interference fragment. All data were presented as mean ± SD, *p < 0.05. GPNMB, glycoprotein nonmetastatic melanoma protein B. [Color figure can be viewed at wileyonlinelibrary.com]

To detect the role of *GPNMB*, we designed the RNAi of *GPNMB*, which can especially suppress the expression of *GPNMB*. As shown in Figure 5A, *GPNMB* interference fragments were successfully transfected in SY5Y cells by CY3. Moreover, qRT‐PCR results revealed that the *GPNMB‐F1* and *GPNMB*‐F3 expressions declined compared with the NC group, which showed that *GPNMB*‐F3 was the most effective interference fragment (Figure 5B, *p* < 0.05).

### 3.6 Change of function after *GPNMB* interference

**Figure 6:**
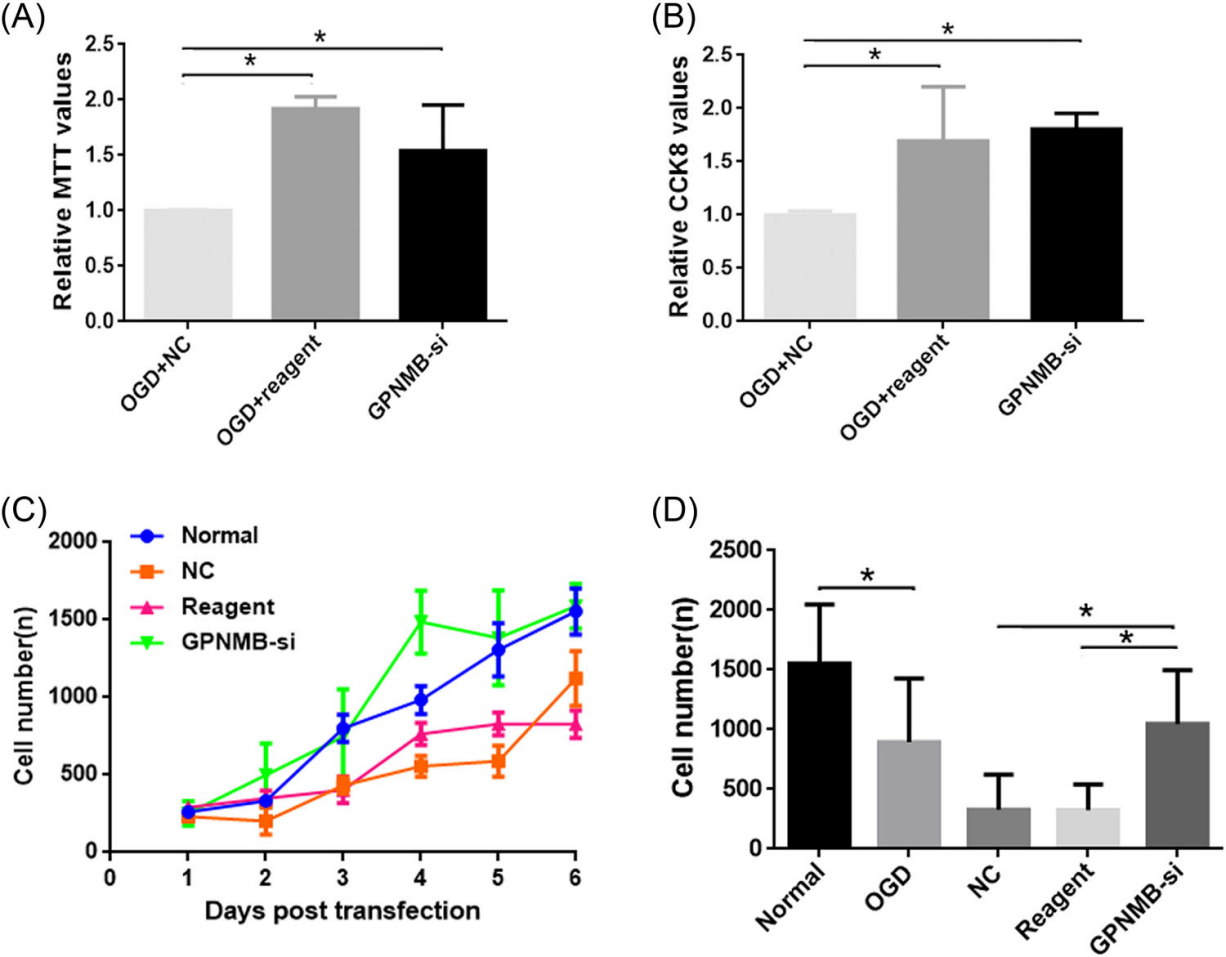
The detection of the *GPNMB* interference function. (A, B) Relative values of MTT and CCK‐8 after interference with *GPNMB* in the OGD + NC, OGD + reagent, and *GPNMB*‐si groups, respectively. (C) Variation trend of the cell growth curve in the normal, NC, reagent, and *GPNMB*‐si groups at 0–6 days posttransfection. (D) The change of cell numbers in the normal and OGD groups, and the NC, reagent, and *GPNMB*‐si groups after OGD injury. All data were presented as mean ± SD, *p < 0.05. CCK‐8, cell counting kit‐8; GPNMB, glycoprotein nonmetastatic melanoma protein B; MTT, methyl thiazolyl tetrazolium; OGD, oxygen–glucose deprivation. [Color figure can be viewed at wileyonlinelibrary.com]

To identify change of function after *GPNMB* interference, MTT and CCK‐8 assays were performed. The results indicated that the relative values of both MTT and CCK‐8 were higher in the OGD + reagent and *GPNMB*‐si groups compared with the OGD + NC group (Figure 6A,B, *p* < 0.05). Moreover, the cell growth curve showed a higher variation trend of the growth curve in the *GPNMB*‐si group compared to the normal group; however, a lower variation trend of the growth curve in the NC and reagent groups was found compared with the normal group (Figure 6C). Furthermore, the results showed that compared to the normal group, the cell number was reduced in the OGD group and higher cell numbers were found in the *GPNMB*‐si group compared with the NC and reagent groups (Figure 6C, *p* < 0.05).

### 3.7 Potential molecular mechanism analysis of *GPNMB* in NHIE: Alternative splicing

**Figure 7:**
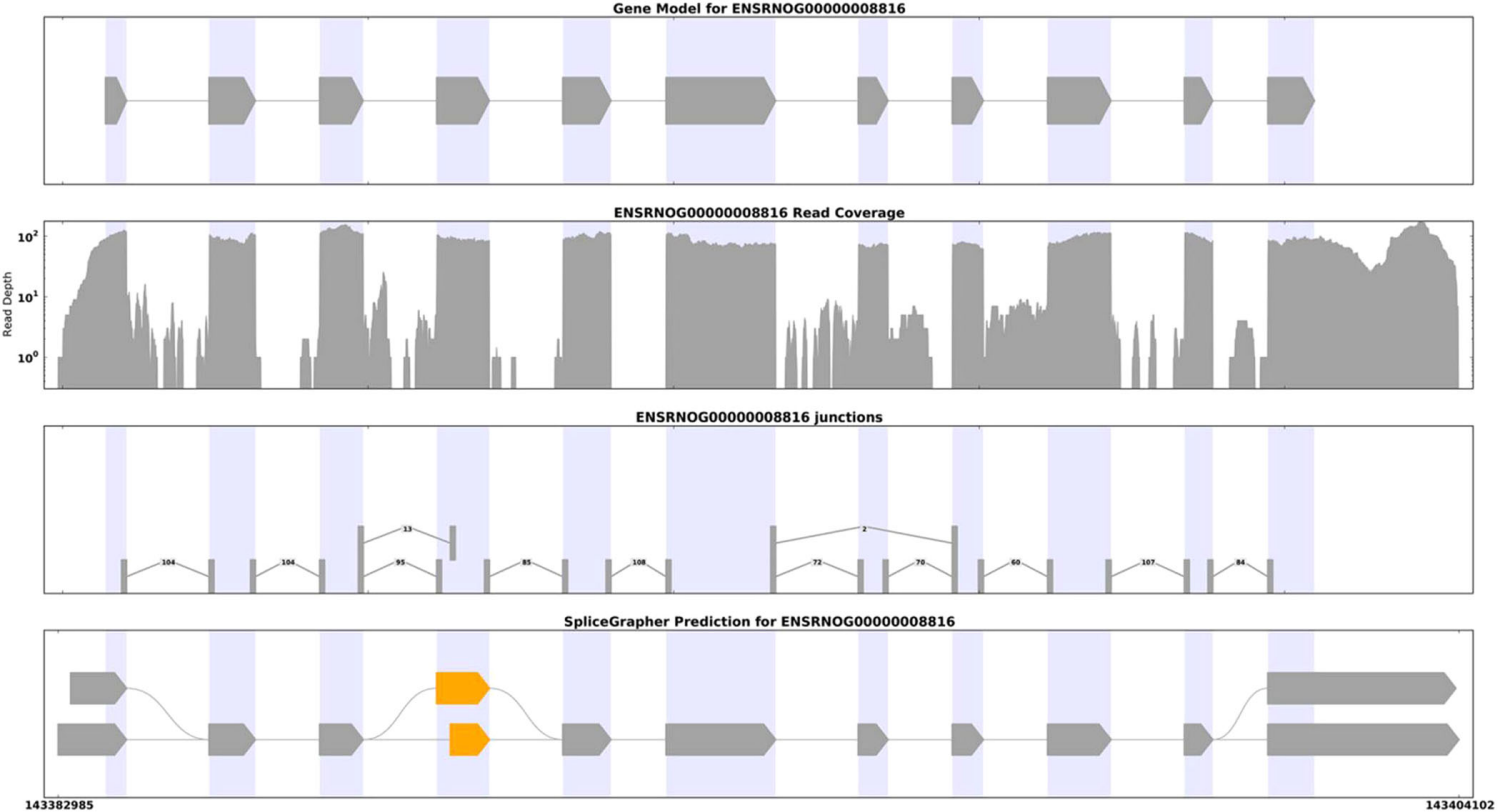
The alternative splicing pattern of the gene. The above picture reflects the splicing of a gene from different angles. From top to bottom, four boxes include the following points: the original splicing model of the gene, different splicing forms with different color markers; the distribution of the reads on the gene; the number of splicing interfaces (splicing junction) and supported mapped reads; the newly predicted splicing event of the gene, with different types of alternative splicing events used for different color markers. [Color figure can be viewed at wileyonlinelibrary.com]

To elucidate the potential molecular mechanism of GPNMB in NHIE, the alternative splicing in *GPNMB* expression was analyzed using Splice Grapher software to predict a new alternative splicing event. We found that the alternative splicing type was the Alternative 3ʹ splice site, and the alternative splicing site was 143382985:143404102 (Figure 7).

## 4 DISCUSSION

In our research, we screened out differentially expressed *GPNMB* post‐HI‐induced injury in vivo, and then we confirmed its expression and explored its function in OGD in vitro. The main result of this study was that the alternative 3′ splice site of *GPNMB* promoted neuronal survival and growth after NHIE injury.

### 4.1 The potential role of *GPNMB* in brain injury

As a result, *GPNMB* was upregulated posthypoxic–ischemic injury in vivo and in vitro, which suggested its potential role in NHIE. *GPNMB* is involved in metabolism, inflammation, cell differentiation, migration, and neuroprotection and has been reported to be related to inflammation, neurodegenerative diseases, and autoimmune diseases.^23^, ^24^, ^25^ It has been reported in the past that *GPNMB* can be detected in the brain. Elevated expressions of *GPNMB* in cerebrospinal fluid in the brain have been considered as possible biomarker candidates for several neurological diseases, such as ALS. Quantitative analysis of cerebrospinal fluid proteins revealed elevated expression of *GPNMB* in patients with short‐lived ALS.^26^, ^27^ In addition, an analysis of human brain samples showed higher expression of *GPNMB* in the substantia nigra in patients with sporadic Parkinson's disease than in healthy controls.^25^ In recent years, increasingly more evidence has shown that *GPNMB* is linked to inflammation and neuroinflammation. Neuroinflammation is one of the markers of nervous system diseases.^23^, ^24^ In an Alzheimer's disease mouse model, *GPNMB* was strongly upregulated in microglia subsets,^24^ suggesting that *GPNMB* may be part of the unique microglial activation state in neurodegenerative diseases. These studies and reports indicate that *GPNMB* may play a potential role in NHIE.

### 4.2 Possible mechanism of *GPNMB* in NHIE

In the present study, we found that interference of *GPNMB* promotes neuronal proliferation after NHIE. It is known that *GPNMB* plays a crucial role in different kinds of neurological diseases. Recent evidence has suggested that *GPNMB*‐si is an influential factor that could inhibit the progression of glioma.^12^, ^28^ Meanwhile, some researchers have reported that *GPNMB* is overexpressed and secreted by breast cancer cells, contributing to breast cancer cell survival,^29^ which indicates that downexpression of *GPNMB* could be considered as a prospective target for treating tumors. Moreover, it has been shown that the *GPNMB* extracellular fragment serves a neuroprotective function. It was found that the alpha subunits of NKA could be potential binding partners of the *GPNMB* extracellular fragment,^30^ which can activate the phosphatidylinositol 3‐kinase/protein kinase B and mitogen‐activated protein kinase/extracellular signal‐regulated kinase pathways through NKA,  indicating that NKA might be potential molecular targets for the treatment of *GPNMB*‐related diseases.^31^, ^32^ It has been shown that under brain injury conditions, siRNA exerts an important regulatory effect on silencing of *GPNMB*.^33^ A previous study demonstrated that siRNA targeting GPNMB alleviated chronic neuropathic pain‐induced sciatic nerve injury in rats.^34^ To elucidate the changes in *GPNMB* alternative splicing, we found the alternative 3′ splice site of *GPNMB* using Splice Grapher software. Though pre‐messenger RNA splicing is likely to cause widespread changes in RNA splicing, further research may identify a target that specifically regulates cell survival pathways. The splicing machinery could likely serve as an attractive therapeutic target due to the antagonistic activities of the two *GPNMB* isoforms; a single manipulation may simultaneously alter the levels of both pro‐ and antiapoptotic proteins, like Bcl‐x.^35^

In conclusion, using the NHIE model in vitro and in vivo, the results demonstrated that *GPNMB* is upregulated in the NHIE model, and its alternative splicing regulation improves neuron survival and growth. The results in our study provide new insight into the mechanism of NHIE, but more studies are needed to verify its effect on NHIE in vivo.

## AUTHOR CONTRIBUTIONS

Rong He and Guo‐Jiao Chen contributed to the central idea of the study, created the figures, and wrote the first draft of the manuscript. Shan‐Shan Yan, Jing‐Han Zhang, and Ji Zhang contributed to conducting of the experiments and carrying out analyses. Rong He, Isaac Bul Deng, and Guo‐Jiao Chen revised the manuscript. All authors have agreed to the final submitted version of the manuscript.

## CONFLICT OF INTEREST

The authors declare no conflict of interest.

## ETHICS STATEMENT

All animal experiments in our research were followed and approved by the Ethics Committee of Kunming Medical University (No. Kmmu2019038) and study included human participants was approved by Ethics Committee of First Affiliated Hospital of Kunming Medical University (Approval No. 2015‐9).

## Data Availability

The data of our study are available upon reasonable request.
